# Editorial: Environmental pollutant and oxidative stress in terrestrial and aquatic organisms

**DOI:** 10.3389/fphys.2022.1073582

**Published:** 2022-11-18

**Authors:** Paola Venditti, Carlos Gravato, Gaetana Napolitano

**Affiliations:** ^1^ Department of Biology, Federico II University of Naples, Naples, Italy; ^2^ Faculdade de Ciências da Universidade de Lisboa and Centro de Estudos do Ambiente e do Mar (CESAM), Lisboa, Portugal; ^3^ Department of Science and Technology, Parthenope University of Naples, Naples, Italy; ^4^ International PhD Programme/UNESCO Chair “Environment, Resources and Sustainable Development”, Department of Science and Technology, Parthenope University of Naples, Naples, Italy

**Keywords:** ROS, oxidative stress, antioxidants, pollutant, environment

This Research Topic aimed to attract articles on the effects of environmental pollutants on the metabolic and redox adaptive responses of both terrestrial and aquatic organisms and on the impact of oxidative stress on the health of living organisms and the environment. For aerobic living beings, using oxygen has represented an evolutive benefit as it has allowed them to obtain large quantities of metabolic energy necessary for vital processes (Napolitano et al., 2022). However, oxygen utilization is accompanied by the production of radicals and other reactive oxygen species (ROS) that can cause damage to cells (Di Meo et al., 2022). For this reason, the development of aerobic life has gone hand in hand with an efficient antioxidant defense system (Napolitano et al., 2021). However, several factors can disturb the delicate balance between ROS production and their elimination, inducing a condition of oxidative stress that can predispose to several diseases (Di Meo et al., 2022). Many environmental pollutants can elicit oxidative stress in aquatic and terrestrial organisms (Leni et al., 2020; Saraiva et al., 2020), and can have detrimental effects on ecosystems and human health.

The topic of the Research Topic has received a fair amount of attention with 6,000 views, and review papers and research articles published between June and October 2022 have already gathered a total of more than 4,800 views. This shows that the theme chosen possesses an immediate impact on what concerns biomarkers, oxidative stress, and implications for human health.

The polychlorinated diphenyl ethers (PCDEs), a class of emerging organic contaminants including 209 homologs widely used as insulating oil, flame retardant, lubricant, and plasticizer, are largely present in the environment and are characterized by the capacity to induce oxidative stress to biota. Ye et al. compared the *in vivo* effects of different PCDE congeners (4-mono-CDE, 4,4′-di-CDE, 3,4,4′-tri-CDE, 3,3′,4,4′-tetra-CDE, and 2,3′,4,4′,5-penta-CDE) on oxidative stress, damage to organelles and cells, and endocrine disruption in zebrafish using an integrated multi-biomarker response analysis complemented with quantitative real-time experiments. Dose-dependent changes in the antioxidant enzyme activities, MDA contents, and vitellogenin (VTG) levels in the liver of zebrafish were observed, also supported by the transcriptional levels of antioxidant genes (*sod1*, *cat1*, and *gpx1a*). Furthermore, tested PCDEs also significantly altered VTG content and related gene *vtg1*, suggesting their potential estrogen-disrupting effects.

Particular attention is also given to the mixture of pollutants in the water of rivers (phosphorus, ammonia, nitrates, and coliforms) probably derived from wastewater discharges and agricultural runoff. Lipid peroxidation, antioxidant enzymes, and the Integrated Biomarker Response were used to determine the potential toxicity of water samples from two rivers of the Mexican Atlantic Slope (Tecolutla and Tuxpan rivers) in the freshwater mollusk *Physella acuta* Sedeño-Díaz and López-López. The increased oxidative stress parameters were contextualized from an ecological point of view, associating the bioassay results with water quality characteristics. The ecological approach adopted allowed authors to show that the battery of biomarkers related to oxidative stress and antioxidant enzymatic response is an efficient early warning sign to identify oxidative stress.

Among pollutants posing many concerns about human and animal health, the textile industry organic dyes are relevant for the higher environmental concentrations due to the release of untreated textile wastewater. Therefore, research effort is focusing attention on efficient and cheap treatments of textile dyes. A good example concerning the knowledge gathered from different perspectives comes from thermophiles that have been shown to be effective in degrading different dyes catalyzed by different thermostable enzymes. The process of microbial degradation of dyes leads to the generation of ROS and subjects cells to oxidative stress but thermophiles, because they optimally grow at extreme conditions, developed better adaptation mechanisms to resist the impacts of oxidative stress Aragaw et al. Thus, bioremediation concerning pollution resulting from dye-containing textile wastewater is increasingly attracting our attention over other treatment techniques.

Very interesting is the critical analysis of oxidative stress as a pivotal player in the impairment of environmental and human health induced by pollutants and, in this view, the protective roles of antioxidants Anetor et al.

Overall, the articles published in our Research Topic highlighted the fact that oxidative stress and antioxidant responses are associated with basic cell functioning. These characteristics make oxidative stress and antioxidant responses excellent “general biomarkers” for practically all classes of contaminants that always require an increase in energy production for detoxification processes (Saraiva et al., 2020). This is extremely important in toxicity tests, for example, in assessing the toxicity of mixtures of pollutants in laboratory or monitoring studies.
